# Broadening our understanding of the genetics of Juvenile Idiopathic Arthritis (JIA): Interrogation of three dimensional chromatin structures and genetic regulatory elements within JIA-associated risk loci

**DOI:** 10.1371/journal.pone.0235857

**Published:** 2020-07-30

**Authors:** Kaiyu Jiang, Haeja Kessler, Yungki Park, Marc Sudman, Susan D. Thompson, James N. Jarvis

**Affiliations:** 1 Department of Pediatrics, Pediatric Rheumatology Research, University at Buffalo Jacobs School of Medicine and Biomedical Sciences, Buffalo, New York, United States of America; 2 Department of Biochemistry, University at Buffalo Jacobs School of Medicine and Biomedical Sciences, Buffalo, New York, United States of America; 3 Genetics, Genomics, & Bioinformatics Program, University at Buffalo Jacobs School of Medicine and Biomedical Sciences, Buffalo, New York, United States of Americass; 4 Center for Autoimmune Genetics & Epigenetics, Cincinnati Children’s Hospital Medical Center, Cincinnati, Ohio, United States of America; 5 Department of Pediatrics, University of Cincinnati College of Medicine, Cincinnati, Ohio, United States of America; University of Iceland, ICELAND

## Abstract

**Objective:**

The risk loci for juvenile idiopathic arthritis (JIA) consist of extended haplotypes that include functional elements in addition to canonical coding genes. As with most autoimmune diseases, the risk haplotypes for JIA are highly enriched for H3K4me1/H3K27ac histone marks, epigenetic signatures that typically identify poised or active enhancers. In this study, we test the hypothesis that genetic risk for JIA is exerted through altered enhancer-mediated gene regulation.

**Methods:**

We mined publically available HiC and other chromatin conformation data to determine whether H3K27ac-marked regions in 25 JIA risk loci showed physical evidence of contact with gene promoters. We also used *in vitro* reporter assays to establish as proof-of-concept the idea that genetic variants in linkage disequilibrium with GWAS-identified tag SNPs alter enhancer function.

**Results:**

All 25 loci examined showed multiple contact sites in the 4 different cell lines that we queried. These regions were characterized by HiC-defined loop structures that included 237 immune-related genes. Using *in vitro* assays, we found that a 657 bp, H3K4me1/H3K27-marked region within the first intron of *IL2RA* shows enhancer activity in reporter assays, and this activity is attenuated by SNPs on the IL2RA haplotype that we identified using whole genome sequencing of children with JIA. Similarly, we identified a 1,669 bp sequence in an intergenic region of the *IL6R* locus where SNPs identified in children with JIA increase enhancer function in reporter assays.

**Conclusions:**

These studies provide evidence that altered enhancer function contributes to genetic risk in JIA. Further studies to identify the specific target genes of genetically altered enhancers are warranted.

## Introduction

Juvenile idiopathic arthritis (JIA) is one of the most common chronic diseases affecting children. JIA is a paradigmatic complex trait, in which there is a known genetic contribution, but the effect sizes at any single risk locus are quite small [1, 2]. However, although the effect conferred by any single risk locus in JIA is small, the net effect of the combined loci is considerable. For example, in a recent study using the Utah Population Database, Prahalad et al found that the relative risk for JIA in siblings was nearly 12-fold that of the broader population (11.6; confidence intervals 4.9–27.5; p<3×10^−8^), and that for first cousins was nearly 6-fold (5.8; confidence intervals 2.5–13.8; p<6×10^−5^) [3]. Thus, understanding the mechanisms through which genetic variants confer risk is critical to our understanding of the pathobiology of JIA. We have recently reported that most of the known JIA-associated risk haplotypes contain functional elements other than genes and are particularly enriched (compared to genome background) for H3K4me1/H3K27ac histone marks, epigenetic signatures associated with enhancer function [4, 5]. JIA is not unique in this regard. Indeed, multiple genome-wide association studies (GWAS) for complex traits performed over the past 12 years have shown that:

(1) most of the tag SNPs that identify risk regions are located in non-coding regions of the genome [6]; and (2) these regions are highly enriched for non-coding elements such as enhancers [7, 8]. The reverse is also true: if one maps enhancer elements in specific cell types, those mapped regions are highly enriched in GWAS-identified SNPs for diseases that affect those particular cells or tissues [9]. This enrichment is significantly greater than that seen at other genomic elements, such as promoters.

Enhancers are non-coding DNA elements that play an important role in regulating gene expression, serving as rheostats that fine-tune gene expression to fit specific physiologic contexts [10]. Enhancers have a characteristic structure that includes the presence of open chromatin bound by multiple transcription factors (TFs) flanked by H3K4me1/H3K27ac-marked boundaries [11]. These TFs form a complex that typically includes p300, mediator, and cohesin, which together facilitate looping and physical contact with the promoters of target genes. In the immune system, enhancers may regulate more than one gene [12] and may not regulate the gene(s) in closest proximity.

The presence of H3K4me1 and H3K27ac and bound TFs at a specific genomic location is not *prima facie* evidence that the region in question has enhancer activity. Therefore, in order to gather additional evidence that JIA risk variants impinge on non-coding, regulatory functions, we interrogated additional chromatin features encompassing the established JIA risk loci. To do this, we took advantage of the fact that chromatin is organized within distinct regions determined by DNA looping structures. These looping structures are referred to as topologically associated domains (TADs) [13]. TAD boundaries are determined by the presence of anchor structures consisting of the transcription regulator, CTCF, and cohesin [13], and serve to facilitate the interactions between specific genomic regions while constraining others [14]. TADs can be identified using non-directed chromatin capture techniques such as HiC [15], which allows for the detection of pairwise contacts between any two genomic locations based on their physical proximity in the 3D genome [16]. These physical interactions can visualized using software programs like *Juicebox* [17] and/or by querying publically available chromatin data via the *3D Genome Browser* [18]. We used this publically available HiC data to query pathologically relevant cell lines, specifically seeking to determine whether we could identify specific regions of contact between putative JIA-associated enhancer regions and promoters of genes within the same TADs as the putative enhancers. For two of these loci, *IL2RA* and *IL6R*, we then used conventional *in vitro* techniques to demonstrate enhancer function and the effects of JIA-associated genetic variants on that function. We chose *IL2RA* because of its known association with a broad spectrum of autoimmune diseases [19–21], including JIA [22], and *IL6R* because this locus does not appear on GWAS for any other autoimmune disease and, thus, its effects appear to be specific for JIA [23].

## Methods

### Defining JIA haplotypes

We examined the JIA haplotypes identified in a recent review article [24] as well as the genetic fine mapping study published by Hinks et al [25]. We identified the linkage disequilibrium (LD) blocks (haplotypes) defined by the index single nucleotide polymorphisms (SNPs) exactly as described in [4]. In brief, we used a SNP Annotation And Proxy search (SNAP) database [26] (http://www.broadinstitute.org/mpg/snap) to define LD blocks based on the location of each SNP using the following settings: SNP dataset: 1000 Genome pilot 1 and HapMap 3 (release 2), r^2^ threshhold: 0.9, Population Panel: CEU, Distance limit: 500. We selected the smallest number as our start location and the largest number as our stop location for each defined LD block.

### Defining TADs

We used the *Juicebox* software program (http://aidenlab.org/juicebox/ [17]) to identify and visualize TADs that encompass the JIA haplotypes and the putative enhancers on those haplotypes. Juicebox annotates publically available third party chromatin data (e.g., HiC) and can also be used by investigators to query their own data. We mined publicly available third party Hi-C data on the Juicebox program to identify TADs in immune cells or immune cell lines that are plausibly relevant to JIA. These data can be accessed on the *Juicebox* software by opening the “Files” tab (top left hand part of the screen) and selecting “HiC Data Archive.”

**Fig 1:**
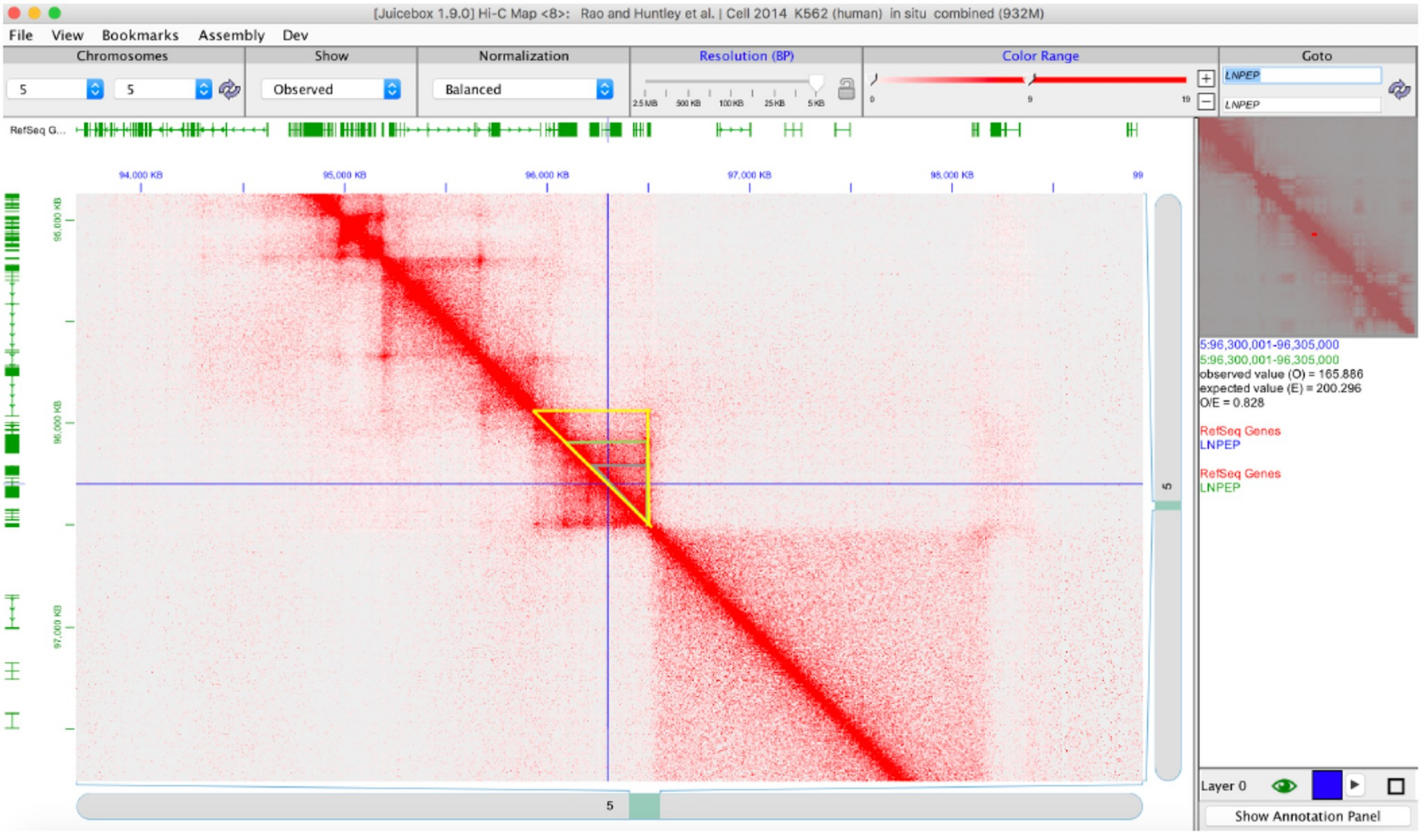
*Juicebox* software visualization of physical chromatin contacts within the IL6R locus. Data are from HiC analysis of unstimulated THP-1 cells. Multiple loops and sub-loops (represented by the grey, green, and yellow triangles) can be identified. The crossed blue lines indicate the position of the H3K4me1/H3K27ac-marked putative intergenic enhancer that we have identified within this locus.

The cell lines from which we analyzed Hi-C data were: GM12787 (B cell lineage [27]), K562 (undifferentiated myeloid lineage [27]), human cord blood CD4+ T cells [28], and THP-1 cells (monocyte lineage [29]). For each cell line, the Hi-C map was normalized to “balanced,” which allowed for correction of experimental bias. While there are multiple algorithms in the *Juicebox* software that remove biases, we used the Knight and Ruiz balanced algorithm as recommended by the authors [17]. To identify TADs associated with specific enhancers, we used a 5KB resolution, which allowed us to visualize chromatin loops as peaks. Using the 1D annotation panel, we selected for the RefSeq genes to allow us to determine the specific genes incorporated within the identified chromatin loop domains. Since *Juicebox* allows users to analyze heat maps visually, TADs are defined by the user, although this approach may lead to inter-observer variation. Our approach is shown in Fig 1. The blue intersecting lines show the “straight edge” tool (which we will refer to as the *cursor*). This tool allows users to pinpoint an exact chromosomal location of a region of interest. For each locus and each cell type, we set the cursor at the center of a putative enhancer, i.e., a region marked by the presence of open chromatin (ENCODE DNase1 data), H3K4me1/H3K27ac histone marks, and abundant transcription factor binding (ENCODE and *Roadmap Epigenomics* data). As seen on the right hand side of Fig 1 in the “information pane”, the cursor is set to a location at chromosome 5:96,300,001–96,305,000. This chromosome location represents the H3K4me1/H3K27ac-marked region within the JIA-associated *LNPEP* locus. The chromosome locations used for each putative enhancer were identified within known JIA risk haplotypes. For this study, we queried 25 JIA haplotypes in which we have identified putative enhancers in both lymphoid and myeloid cells [4, 5]. These regions are shown in Table 1. In the Juicebox software, TADs are visualized as boxes, as shown in Fig 1. Note that more than one loop structure can be identified in many functionally active chromatin regions, as has been seen in multiple cell types [30].

**Table 1:** JIA risk haplotypes queried In HiC analyses.

LD region	SNP	Location	Adjacent Gene
chr12: 111884608–111932800	rs3184504	Intronic	*SH2B3*
chr12: 112486818–112906415	rs17696736	Intronic	*NAA25*
chr18: 12821903–12880206	rs7234029	Intronic	*PTPN2*
chr2: 191900449–191935804	rs3821236	Intronic	*STAT4*
chr22: 24234493–24237862	rs755622	Last exon	*MIF*
chr3: 119125202–119243934	rs4688013	Intronic	*TIMMDC1*
chr3: 119125202–119243934	rs4688011	Intronic	*TIMMDC1*
chr3: 46253650–46350716	rs79893749	Intronic	*CCR1*
chr4: 123141054–123548068	rs17388568	Intronic	*ADAD1*
chr6: 112359543–112448654	rs2280153	Intronic	*WISP3*
chr7: 28152193–28243473	rs10280937	Intronic	*JAZF1*
chr7: 28152193–28243473	rs73300638	Intronic	*JAZF1*
chr9: 123636121–123723351	rs10818488	Intergenic	*TRAF1-C5*
chr9: 123640500–123706382	rs2900180	Intergenic	*TRAF1-C5*
chr1: 114303808–114377568	rs6679677	Intergenic	*PHTF1-RSBN1*
chr1: 154291718–154379369	rs11265608	Intergenic	*IL6R*
chr1: 154291718–154379369	rs72698115	Intronic	*IL6R*
chr 10: 6078553–6097283	rs7909519	Intronic	*IL2RA*
chr 10: 90759613–90764891	rs7069750	Intronic	*FAS*
chr 12: 111884608–111932800	rs7137828	Intronic	*ATXN2*
chr 14: 69250891–69260588	rs12434551	Intergenic	*ZFP36L1-RAD51B*
chr 14: 69250891–69260588	rs3825568	Exonic	*ZFP36L1*
chr 5: 96220087–96373750	rs27290	Intronic	*LNPEP*
chr 5: 96220087–96373750	rs27293	Intronic	*LNPEP*
chr 5: 131813219–131832514	rs4705862	Intergenic	*C5orf56-IRF1*

Direct evidence of physical interaction between H3K27ac-marked regions within the JIA haplotypes and promoters of multiple genes within the TADs defined in the previous analysis was undertaken by mining promoter capture HiC data available on the 3D Genome Browser (http://promoter.bx.psu.edu/hi-c/ [18, 31]). This site also queries and annotates a broad range of publicly available, third party chromatin data For these studies, we queried data from total CD4+ T cells as reported by [31], which can be found by selecting the blue “Capture Hi C” tab on the top middle tab of the opening screen, selecting “Tissue,” and choosing “CD4_Total” from the drop-down menu.

### Gene ontology analysis

We sought to determine whether there were common functions for the genes within the TADs identified from our analysis of HiC data. We performed these analyses for one lymphoid (K562) and one myeloid (THP-1) cell line. We uploaded the genes from each of these cell lines into the publically available GOrila software suite [32] (http://cbl-gorilla.cs.technion.ac.il) using expressed genes in each cell line as background. The list of expressed genes used as background for these studies was curated from the *Harmonizome* data base [33] (http://amp.pharm.mssm.edu/Harmonizome/). We then used the publically available REVIGO software [34] (http://revigo.irb.hr) to eliminate redundant GO terms and to provide easy analysis of the results.

### Experimental verification of enhancer function within a JIA haplotype

**Fig 2:**
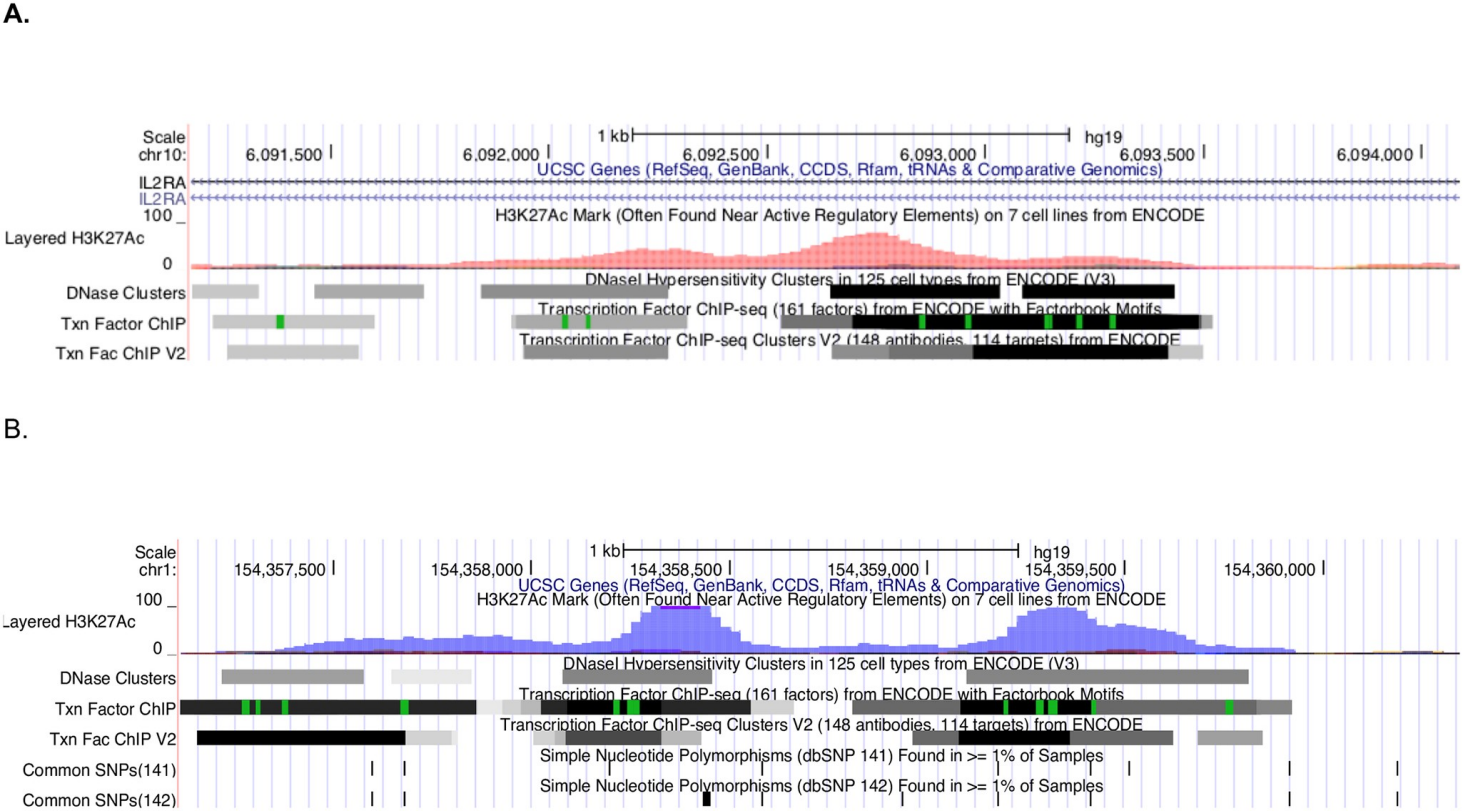
UCSC genome browser view of a select region within the *IL2RA* haplotype within the first intron of the *IL2RA* (A) gene and a select region within the *IL6R* haplotype (B). These regions are characterized by dense H3K27ac histone peaks (shown as magenta/purple peaks-ENCODE and *Roadmap Epigenomics* data). The black and gray horizontal lines represent DNAse hypersensitive sites and TF binding from ChIPseq data (also from ENCODE and *Roadmap Epigenomics* data). Identical chromatin features are seen in this region in both human CD4+ T cells and neutrophils.

In order to test the validity of the computational analysis of the HiC data, we performed *in vitro* experiments to examine putative enhancer function within 2 loci: *IL2RA*, a locus common to JIA [25] and multiple other autoimmune diseases [20, 35–37], and *IL6R*, a locus that is unique to JIA [38], as noted in the introduction. We used H3K4me1/H3K27ac ChIPseq data from Roadmap Epigenomics (accession numbers GSM1220567, GSM1220560 and GSM1102805) as well as our own H3K4me1/H3K27ac ChIPseq data in neutrophils (GSE66896) [4] to identify a 1500 bp region within the first intron of *IL2RA* that was likely to have functional activity (Fig 2A) as well as a 1,669 bp intergenic region upstream of the *IL6R* gene with the same features (Fig 2B). This region has prominent H3K4me1 and H3K27ac marks in both cell types as well as abundant transcription factor binding sites (ENCODE and Roadmap Epigenomics data). Subsequent studies were focused on 657bp (chr10:6092661–6093317) within the *IL2RA* region as well as well as chr1:154357931–154359600 in the *IL6R* region.

### Purification and amplification of DNA by Polymerase Chain Reaction (PCR)

DNA purification from whole blood was performed using DNeasy Blood & Tissue Kit (Qiagen, USA). The purified DNA was used directly in the PCR. All PCR amplifications mixtures were carried out in a total volume of 50 μL containing 25 μL NEBNext^®^ High-Fidelity 2X PCR Master Mix (New England BioLabs, Ipswich, MA, USA), 80 ng genomic DNA as a template, 50 pmol final concentrations from each of the forward and reverse primers, and the volume was completed to 50 μL using nuclease-free sterile water. The oligonucleotide sequences for cloning were FP: 5′- GTGGCCATGAGGAATCTGTAAG -3′ and RP: 5′- CGACAATGAAACCAGGCAGA -3′ and the product size was 539bp (chr10:6092661–6093199); FP: 5′- CAGGTCCTCTTGCACTAGATTG -3′ and RP: 5′- AAGTTGACGTCAGCCTCTTC-3′ and the product size was 533bp (chr10:6092785–6093317); FP: 5′- GAGGTGATGTGTTCTTCTCATCT -3 and RP: 5′ - TCATTTCTTCCTTGCTGTCCT-3′ and the product size was 402bp (chr10:6089866–6090267); FP: 5′- GTGGCCATGAGGAATCTGTAAG -3′ and RP: 5′- AAGTTGACGTCAGCCTCTTC-3′ and the product size was 657bp (chr10:6092661–6093317); FP: 5′- GGTGAAGGGATTTCAGCCTTAT-3′ and RP: 5′- AGGAGAACAGGAGGACACAT-3′ and the product size was 594bp (chr1:154357931–154358524); FP: 5′- TCAGCTGTCTGGTGATCTTTG-3′ and RP: 5′-GGCTTGTAAACTCGGGAACT-3′ and the product size was 647bp (chr1:154358954–154359600). XhoI (5′-C^TCGAG-3′) and BglII(5′-A^GATCT-3′) restriction endonuclease recognition sites were incorporated in the oligonucleotides for directional cloning.

### Cloning of the IL2RA and IL6R enhancers

The PCR amplified fragments were digested using XhoI and BglII (New England Biolabs, MA, USA) and ligated into a pGL4.23[*luc2*/minP] vector (Promega E8411, Madison, MI, USA), previously digested using the same endonucleases. After an overnight incubation at 16°C, the ligation mixtures were transformed into competent *E*. *coli* JM109 cells (Promega). Plasmid DNA was isolated by Miniprep kit (Qiagen) according to the manufacturer’s instructions. All recombinant plasmid sequences were confirmed by Sanger sequencing.

### Site-directed mutagenesis

Mutations were introduced with the QuikChange II Site-Directed Mutagenesis Kit (Agilent, Santa Clara, CA). The primers were designed according to the manufacturer’s protocol, utilizing the QuikChange Primer Design Tool (http://www.genomics.agilent.com). The wild-type (wt) construct pGL4.23-IL2RA enhancer (chr10:6092661–6093317) was used as template to generate 4 enhancer genetic variants. The primer oligonucleotide sequences were: rs117119468, chr10:6092989, C>T, FP: 5'-AATAAAAGAGCCATCTCTCCAGACATCTGTCTGACG-3’, RP: 5'-CGTCAGACAGATGTCTGGAGAGATGGCTCTTTTATT-3’; rs12722502, chr10: 6093139, C>T, FP: 5'-GTGGTCTCTGCTTCCAGTCCTTGTGGTAGCA-3’, RP: 5'-TGCTACCACAAGGACTGGAAGCAGAGACCAC-3’; rs370928127,chr10: 6092686, A>G, FP: 5'-TGCGTGCTCTGCTTCTGCGATCTTACAGATTCCTC-3’, RP: 5'-GAGGAATCTGTAAGATCGCAGAAGCAGAGCACGCA-3’; rs552847047, chr10: 6093178, G>T, FP: 5'-GAAACCAGGCAGAGACTGCAACCCAGCTG-3’ and RP: 5'-CAGCTGGGTTGCAGTCTCTGCCTGGTTTC-3’.

The wild-type (wt) construct pGL4.23-IL6R enhancers (chr1:154357931–154358524 and chr1:154358954–154359600) were used as template to generate 2 enhancer genetic variants, respectively. The primer oligonucleotide sequences were: rs540546266, chr1:154358009, C>T, FP: 5'-GCCACTGAGTTGAGGTGGGGCTGGC-3’, RP: 5'-GCCAGCCCCACCTCAACTCAGTGGC-3’; rs532200576, chr1:154358496, T>C, FP: 5'-GAGGACACATGAGTTTGGGGCTTCATACAAACGTGTG-3’, RP: 5'-CACACGTTTGTATGAAGCCCCAAACTCATGTGTCCTC-3’; rs9651053, Chr1:154359411, G>A, FP:5'-AGACATGTGGGGACTTCCCCAGCAAACAGTC-3', RP: 5'-GACTGTTTGCTGGGGAAGTCCCCACATGTCT-3' and rs540215820, chr1:154359522, C>T, FP: 5'-CTCTGCCTCCTTTCAGGGGCCAGAACACT-3', RP: 5'-AGTGTTCTGGCCCCTGAAAGGAGGCAGAG-3'.

#### Luciferase assays

Cells were transiently transfected using electroporation following the protocol of the manufacturer (Nucleofector Device with Cell line kit V, Lonza, USA). Cells (2x10^6^) were tranfected with 1ug of the pGL4.23-constructs plus 1 ug of the control Renilla plasmid DNA (pRL-TK; Promega) to normalize for transfection efficiency. Twenty-four hours after transfection, the cells were harvested, lysed and analyzed for luciferase activity. Luminescence was measured with the Dual-Luciferase^®^ Reporter Assay System (Promega) in a microplate luminometer (Biotek Cytation 5MV Microplate Reader, Biotek). Results from each construct and from the empty vector were normalized by dividing the luciferase reporter activity by the renilla reporter activity. Relative luciferase activities were calculated by dividing each normalized construct by the normalized empty vector. Relative luciferase activities are representative of at least three independent transfection experiments. Student’s t test was used to determine statistical significance.

## Results

### 3D chromatin structure encompassing the JIA haplotypes

We queried 25 of the JIA-associated haplotypes in which we had previously identified chromatin features associated with enhancer function in both lymphoid and myeloid cells. These common enhancers are likely to regulate important leukocyte developmental and cellular processes.

We identified at least one HiC-defined chromatin loop in each of the cell lines we queried. As expected, the loops demonstrated cell-type specificity, although there were shared loop structures in each of the cell lines we queried. These results are shown in Table 2, which provides the chromatin coordinates for each TAD as well as those of the JIA-associated the LD blocks in which those TADs are located. The presence of contact loops within each of the queried cell types at each of the queried positions provides additional support to the idea that these regions contain at least one active enhancer. Furthermore, they support the idea that genetic risk may be exerted on both the innate and adaptive immune systems.

**Table 2:** Identification of TADs incorporating JIA risk haplotypes within human leukocyte cell lines.

Enhancer	Location of LD Block	GM12878 TADs Location	K562 TADs Location	T-Cell TADs Location	THP-1 Macrophage TADs Location
SH2B3	chr12: 111884608–111932800	chr12: 111,830,001–112,125,000 chr12: 111,770,001–112,125,000	chr12: 111,835,001–112,110,000 chr12: 111,760,001–112,110,000	chr12: 111,730,001–112,965,000	chr12: 111,755,001–111,970,000 chr12: 111,885,001–112,185,000
NAA25	chr12: 112486818–112906415	chr12: 112,450,001–112,845,000 chr12: 112,380,001–112,845,000	chr12: 112,255,001–112,835,000	chr12: 111,730,001–112,965,000	chr12: 112,250,001–112,850,000 chr12: 111,895,001–112,850,000
PTPN2	chr18: 12821903–12880206	chr18: 12,705,001–12,955,000 chr18: 12,705,001–13,165,000 chr18: 12,385,001–12,955,000	chr18: 12,705,001–12,950,000 chr18: 12,705,001–13,180,000 chr18: 12,380,001–12,945,000	chr18: 12,680,001–12,945,000 chr18: 12,680,001–13,795,000 chr18: 12,395,001–13,795,000	chr18: 12,670,001–12,970,000 chr18: 12,670,001–13,200,000
STAT4	chr2: 191900449–191935804	chr2: 191,650,001–191,920,000 chr2: 191,900,001–192,040,000 chr2: 191,900,001–192,410,000	chr2: 191,910,001–192,400,000	chr2: 191,905,001–192,400,000	chr2: 191,895,001–192,035,000 chr2: 191,895,001–192,385,000 chr2: 191,645,001–192,035,000
MIF	chr22: 24234493–24237862	chr22: 24,200,001–24,675,000 chr22: 23,860,001–24,245,000	chr22: 23,870,001–24,260,000 chr22: 24,190,001–24,700,000	chr22: 23,885,001–24,305,000	chr22: 24,195,001–24,695,000 chr22: 23,865,001–24,295,000
TIMMDC1	chr3: 119125202–119243934	chr3: 119,185,001–119,415,000 chr3: 118,990,001–119,285,000 chr3: 118,950,001–119,415,000	chr3: 118,600,001–119,400,000	chr3: 118,595,001–119,405,000	chr3: 119,190,001–119,410,000 chr3: 118,955,001–119,410,000 chr3: 118,600,001–119,410,000
CCR1	chr3: 46253650–46350716	chr3: 46,240,001–46,435,000 chr3: 46,130,001–46,435,000 chr3: 45,975,001–46,435,000 chr3: 45,795,001–46,435,000	chr3: 46,250,001–46,420,000 chr3: 46,125,001–46,420,000	chr3: 46,130,001–46,510,000 chr3: 45,785,001–46,805,000	chr3: 46,240,001–46,420,000 chr3: 46,240,001–46,490,000 chr3: 46,120,001–46,490,000 chr3: 45,975,001–46,490,000
ADAD1	chr4: 123141054–123548068	chr4: 122,800,001–123,505,000	chr4: 123,225,001–123,500,000 chr4: 122,815,001–123,500,000	chr4: 122,675,001–12,357,000	chr4: 123,230,001–123,495,000 chr4: 123,230,001–123,855,000 chr4: 122,810,001–123,855,000
WISP3	chr6: 112359543–112448654	chr6: 112,015,001–112,380,000 chr6: 111,805,001–112,495,000 chr6: 111,575,001–112,380,000	chr6: 112,015,001–112,485,000	chr6: 112,020,001–112,385,000	chr6: 112,370,001–112,490,000 chr6: 112,030,001–112,490,000
JAZF1	chr7: 28152193–28243473	chr7: 28,105,001–28,425,000 chr7: 28,185,001–28,900,000	chr7: 28,085,001–28,945,000 chr7: 27,220,001–28,160,000	chr7: 27,235,001–28,210,000 chr7: 28,220,001–28,945,000	chr7: 28,140,001–28,310,000
TRAF1-C5	chr9: 123636121–123723351	chr9: 123,510,000–123,840,000 chr9: 123,510,001–123,965,000 chr9: 123,510,001–124,165,000	chr9: 123,515,001–123,840,000	chr9: 123,510,001–123,980,000	chr9: 123,600,001–123,720,000 chr9: 123,510,001–123,830,000 chr9: 123,380,001–123,830,000
PHTF1-RSBN1	chr1: 114303808–114377568	chr1: 114,235,001–114,530,000	chr1: 114,225,001–114,515,000	chr1: 114,315,001–114,540,000 chr1: 113,650,001–114,335,000	chr1: 114,295,001–114,520,000 chr1: 113,680,001–114,520,000
IL6R	chr1: 154291718–154379369	chr1: 154,305,001–154,460,000 chr1: 154,245,001–154,535,000 chr1: 153,960,001–154,535,000	chr1: 154,310,001–154,455,000 chr1: 154,310,001–154,530,000 chr1: 154,150,001–154,530,000	No TADs	chr1: 154,285,001–154,450,000 chr1: 154,240,001–154,530,000 chr1: 154,155,001–154,530,000
IL2RA	chr10: 6078553–6097283	chr10: 5,670,001–6,135,000 chr10: 5,480,001–6,455,000	chr10: 5,960,001–6,150,000 chr10: 5,980,001–6,445,000 chr10: 5,665,001–6,135,000	chr10: 6,090,001–6,455,000	chr10: 5,980,001–6,145,000 chr10: 5,980,000–6,450,000
FAS	chr10: 90759613–90764891	chr10: 90,685,001–91,010,000 chr10: 90,590,001–91,010,000 chr10: 90,325,001–91,010,000	chr10: 90,775,001–90,980,000 chr10: 90,685,001–90,980,000 chr10: 90,580,001–90,980,000	chr10: 90,550,001–90,990,000	chr10: 90,695,001–90,790,000 chr10: 90,695,001–90,980,000 chr10: 90,580,001–90,980,000
ATXN2	chr12: 111884608–111932800	**1KB resolution** chr12: 111,833,001–111,896,000 chr12: 111,882,001–112,124,000	chr12: 111,835,001–112,115,000 chr12: 111,760,001–112,115,000	chr12: 111,720,001–112,960,000	chr12: 111,745,001–111,970,000 chr12: 111,890,001–112,185,000
ZFP36L-RAD51B	chr14: 69250891–69260588	chr14: 68,710,001–69,290,000 chr14: 68,410,001–69,290,000 chr14: 68,290,001–69,290,000	chr14: 68,715,001–69,300,000 chr14: 68,405,001–69,300,000 chr14: 68,300,001–69,300,000	chr14: 68,685,001–69,340,000 chr14: 68,400,001–69,340,000	chr14: 68,705,001–69,285,000 chr14: 68,410,001–69,285,000
LNPEP	chr5: 96220087–96373750	chr5:96,080,001–96,510,000 chr5: 95,955,001–96,510,000	chr5: 96,200,001–96,510,000 chr5: 96,085,001–96,510,000 chr5: 95,950,001–96,510,000	chr5: 96,070,001–96,520,000	chr5: 96,085,001–96,510,000 chr5: 95,940,001–96,510,000 chr5: 95,675,001–96,510,000
C5orf56-IRF1	chr5: 131813219–131832514	chr5: 131,715,001–131,855,000 chr5: 131,715,001–132,220,000 chr5: 131,395,001–131,845,000	chr5: 131,710,001–131,865,000 chr5: 131,510,001–131,840,000 chr5: 131,405,001–131,840,000 chr5: 131,710,001–132,240,000	chr5: 131,715,001–132,180,000	chr5: 131,720,001–131,865,000 chr5: 131,720,001–132,215,000 chr5:131,515,001–131,845,000 chr5: 131,390,001–131,845,000

### Gene Ontology (GO) analysis of genes within HiC-defined TADs

We next examined the genes that were situated within the contact loops in each of the cell types that we queried. Each of the loops contained multiple genes, as summarized in S1 Table. In all, we identified 237 genes that are incorporated within the loop structures that encompass the JIA risk haplotypes.

**Fig 3:**
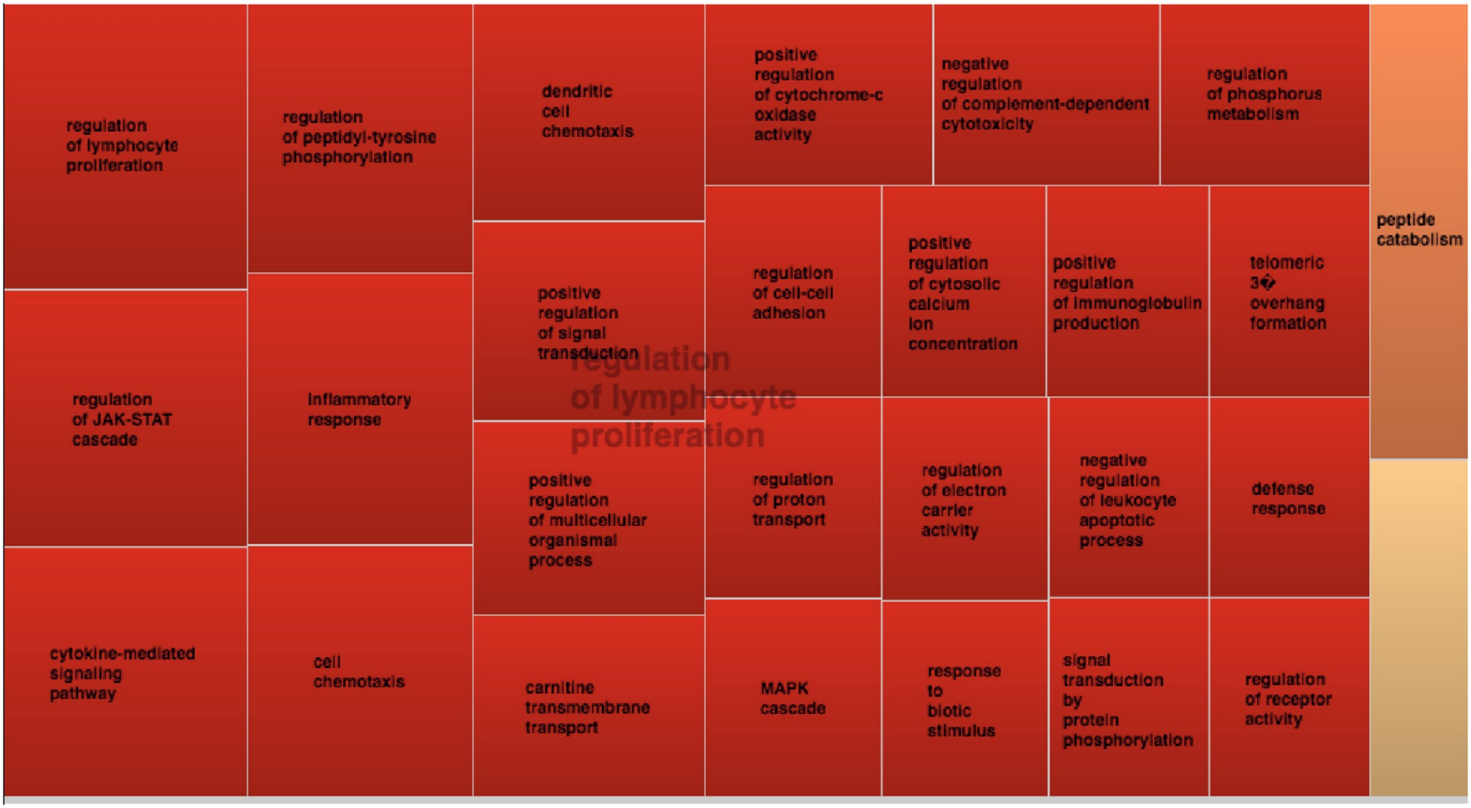
Treemap showing results from REVIGO analysis of gene ontology data derived from analysis of genes within the TADs that incorporate 25 JIA risk loci. Results are from K562 cells. Each rectangle of the treemap indicates a GO terms cluster. Subclusters (related GO terms) of same color are joined into super-clusters marked by centralized semi-transparent text. The size of a rectangle reflects statistical significance p-values of GO terms, with larger rectangles assigned to terms with smaller p-values.

GO analysis combined with REVIGO analysis revealed common themes among these genes within the TADs that incorporated the JIA risk haplotypes. The major GO categories identified among the genes identified in the analysis of HiC data were processes related to lymphocyte activation and proliferation, as shown in Fig 3. The GO terms for “peptide catabolism” also emerged from this analysis, an interesting finding given that this process precedes antigen presentation. We provide supplementary tables showing p-values for GO categories and the number genes in each GO category THP-1 cells (S2 Table) and K562 cells (S3 Table).

### Corroboration of chromatin data: The *IL2RA* and *IL6R* loci include enhancers that are active in both lymphoid and myeloid cells

**Fig 4:**
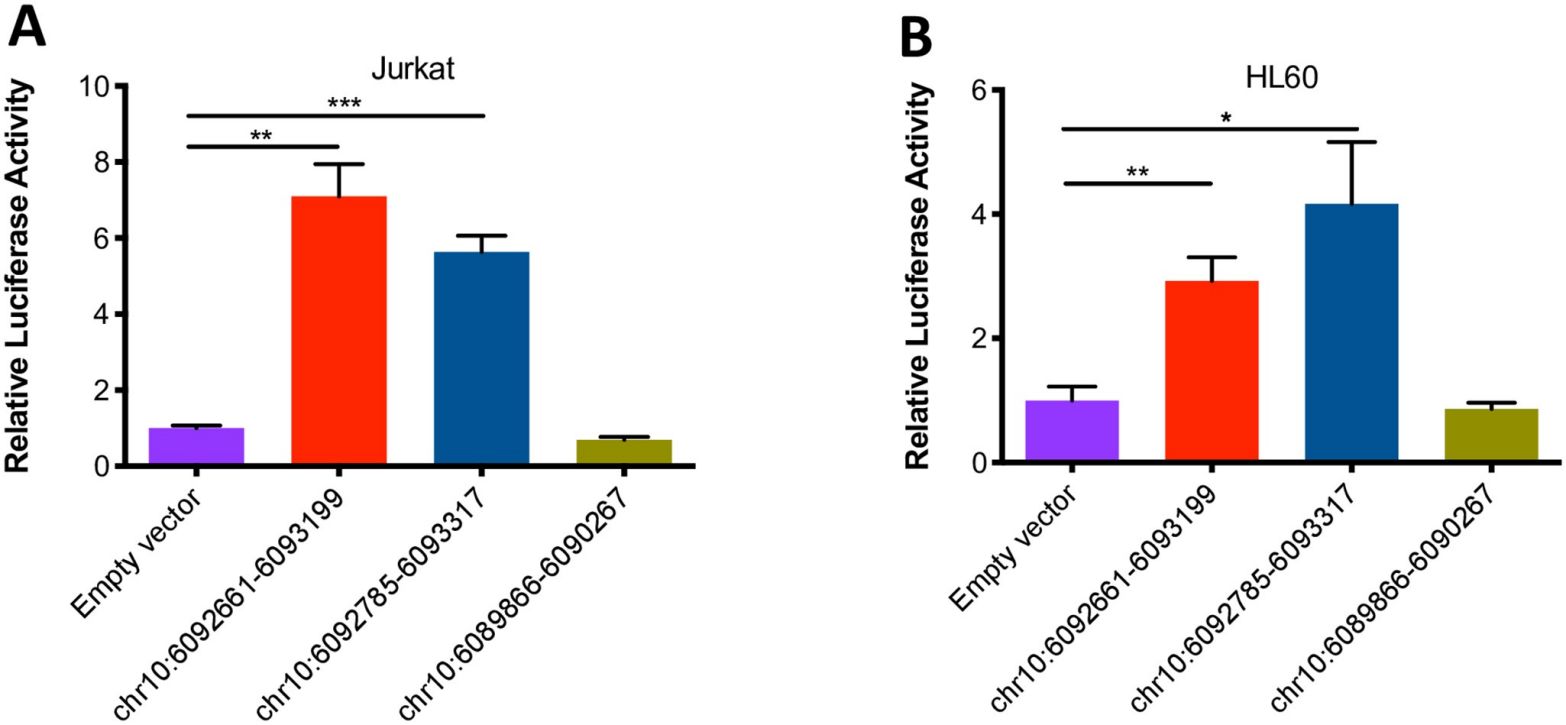
Summary of results from luciferase assays assessing enhancer activity at the *IL2RA* locus in Jurkat T cells and HL60 cells. A and B. The results are from 539bp and 533 constructs, respectively, with the constructs tiled across an H3K4me1/H3K27ac-marked region within JIA-associated haplotype in the first intron of the *IL2RA* gene. Chromosome coordinates for each construct are indicated beneath each bar. Both constructs showed enhancer activity in Jurkat T cells (A) and in HL-60 cells (B). Note that the bar on the far right shows results from the region that includes the index SNP rs7909519. This region does not show enhancer activity. The empty vector (negative control) is the basic pGL4.23 vector (which contains the SV40 promoter but is inefficient at driving luciferase expression). Data are represented as mean ± SEM of 3 (A) or 4 (B) biological replicates, and p values are calculated using Student’s t test. *p ≤ 0.05; ** p≤0.01; ***p≤0.001.

**Fig 5:**
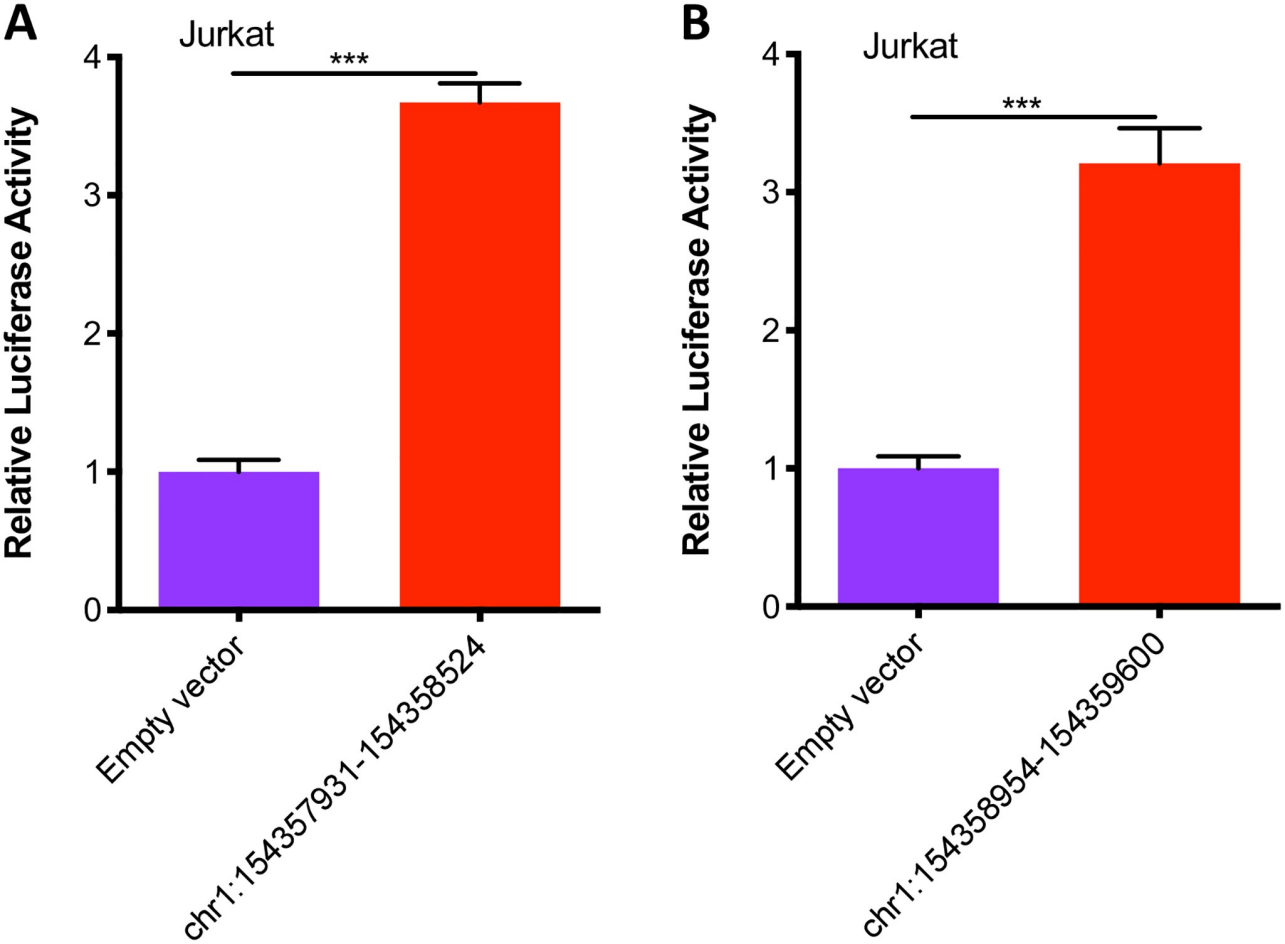
Summary of results from luciferase assays assessing enhancer activity at the *IL6R* locus in Jurkat T cells. A and B. The results are from 594bp and 647bp constructs, respectively, with the constructs tiled across an H3K4me1/H3K27ac-marked region within JIA-associated haplotype in *IL6R* region. Chromosome coordinates for each construct are indicated beneath each bar. Both constructs showed enhancer activity in Jurkat T cells. The empty vector (negative control) is the basic pGL4.23 vector (which contains the SV40 promoter but is inefficient at driving luciferase expression). Data are represented as mean ± SEM of 6 biological replicates, and p values are calculated using Student’s t test. ***p≤0.001.

In order to corroborate the analysis derived from HiC data, we used a reporter assay to assess enhancer function within a 657 bp region within the first intron of the *IL2RA* gene as described in the *Methods* section. We tiled two constructs ((chr10:6092661–6093199, 533bp and chr10:6092785–6093317, 539bp) across the region of interest and tested each construct for luciferase production. Both constructs with brisk enhancer activity that was observable in Jurkat cells as well as myeloid HL60 cells. These results are summarized in Fig 4A and 4B. We also assessed the enhancer function for an intergenic region upstream of the *IL6R* gene (on JIA risk haplotype) that exhibited H3K4me1 / H3K27ac peaks and abundant TF binding (ENCODE and Roadmap Epigenomics data). We tested two constructs (chr1:154357931–15435852, 594bp and chr1:154358954–154359600,647bp) in Jurkat cells. Each construct showed brisk enhancer activity, as shown in Fig 5A and 5B.

### JIA-associated genetic variants alter enhancer function within the *IL2RA* locus and *IL6R* loci

**Fig 6:**
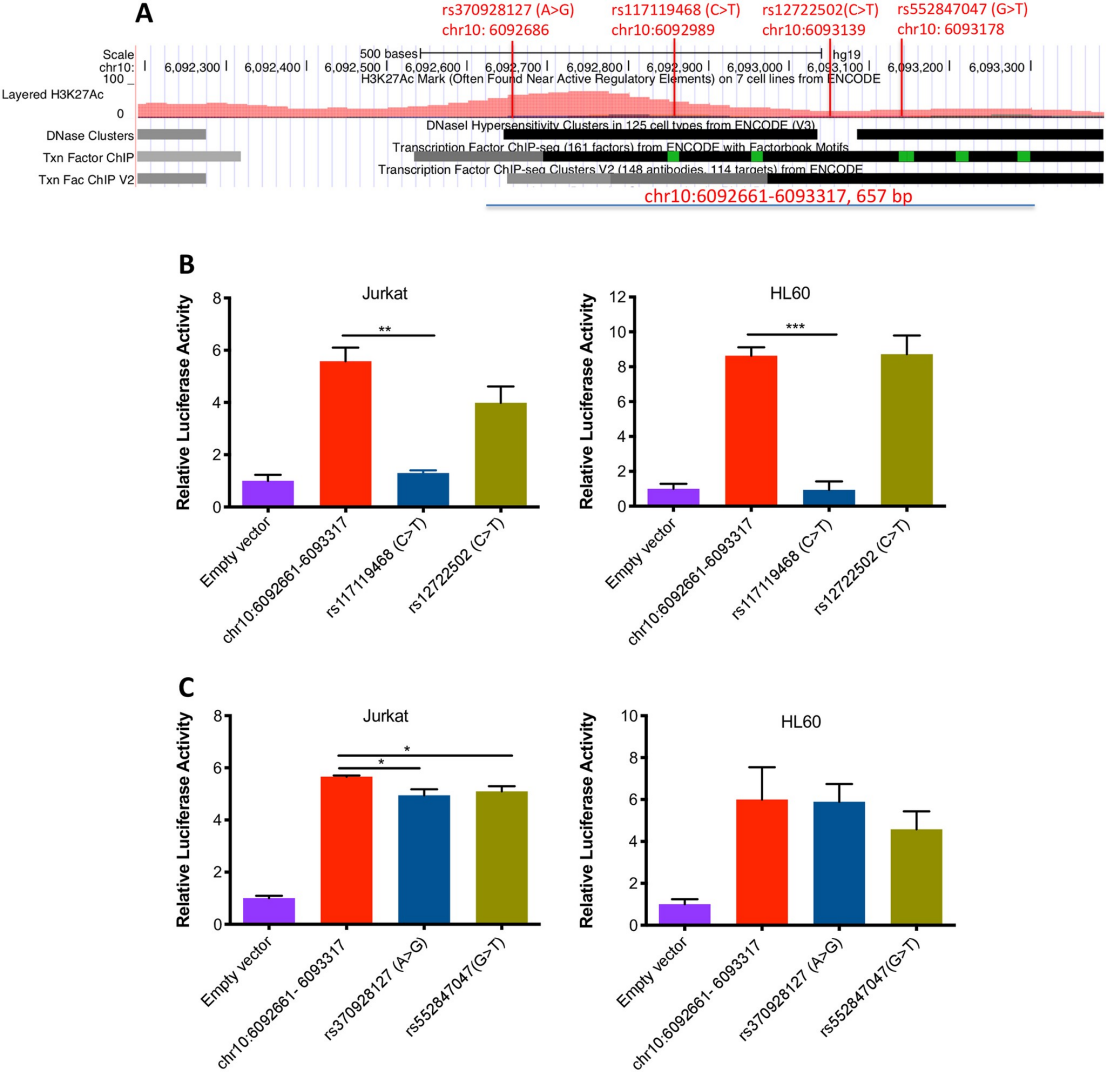
The effects of JIA-associated genetic variants *(IL2RA* locus) on enhancer activity in Jurkat T cells and in HL60 cells. A. The construct within 657 bp region (chr10:6092661–6093317) within the first intron of the *IL2RA* gene was used as template to generate 4 enhancer genetic variants. Of the 4 variants, rs117119468 (C->T), and rs12722502 (C->T) had identified on whole genome sequencing (WGS) of a small number of children with polyarticular JIA [39]; rs370928127 (A>G) and rs552847047 (G>T) were common genetic variants annotated in the 1000 Genomes Project data. B. The second bar represents results from the region queried within a 657 bp region within the first intron of the *IL2RA* gene. This variant, rs117119468 (C->T), almost completely abolishes enhancer function in Jurkat T cells and in HL60 cells (third bar). A second variant, rs12722502 (C->T), reduced luciferase production in Jurkat cells but not in HL60 cells (fourth bar). C. Bar graph showing the effects of two additional variants annotated in the 1000 Genomes Project data, rs370928127 (A>G) and, rs552847047 (G>T), on enhancer activity in Jurkat T cells and in HL60 cells. The third and forth bars show these two variants have an effect on enhancer activity in Jurkat but not in HL60 cells. The empty vector (negative control) is the basic pGL4.23 vector (which contains the SV40 promoter but is inefficient at driving luciferase expression). Data are represented as mean ± SEM of 3 biological replicates, and p values are calculated using Student’s t test. *p ≤ 0.05; ** p≤0.01; ***p≤0.001.

We next sought to test the effects of genetic variants within the identified enhancer in order to demonstrate the idea that genetic risk in JIA may be mediated by alterations in enhancer function. For these experiments, we first tested 2 variants within the *IL2RA* intronic enhancer, rs117119468 (C->T), and rs12722502 (C->T) (Fig 6A), that we had identified on whole genome sequencing (WGS) of a small number of children with polyarticular JIA [39]. These variants were seen only in our JIA cohort and had not previously been annotated in the 1000 Genomes project data. For these experiments, we generated one construct across the region with demonstrated enhancer function (chr10:6092661–6093317, 657bp, Fig 6A). The construct was used as template to generate genetic variants using site-directed mutagenesis as described in the *Methods* section. One variant, rs117119468, completely abolished enhancer function within this locus in Jurkat T cells and in HL60 cells. Another, rs12722502, reduced function by 30% in Jurkat but had no effect in HL60 cells. These results are shown in Fig 6B. We also tested two common genetic variants annotated in the 1000 Genomes Project data rs370928127 (A>G) and rs552847047 (G>T), (Fig 6A). These two variants reduced function by 10% in Jurkat but had no effect in HL60 cells (Fig 6C).

**Fig 7:**
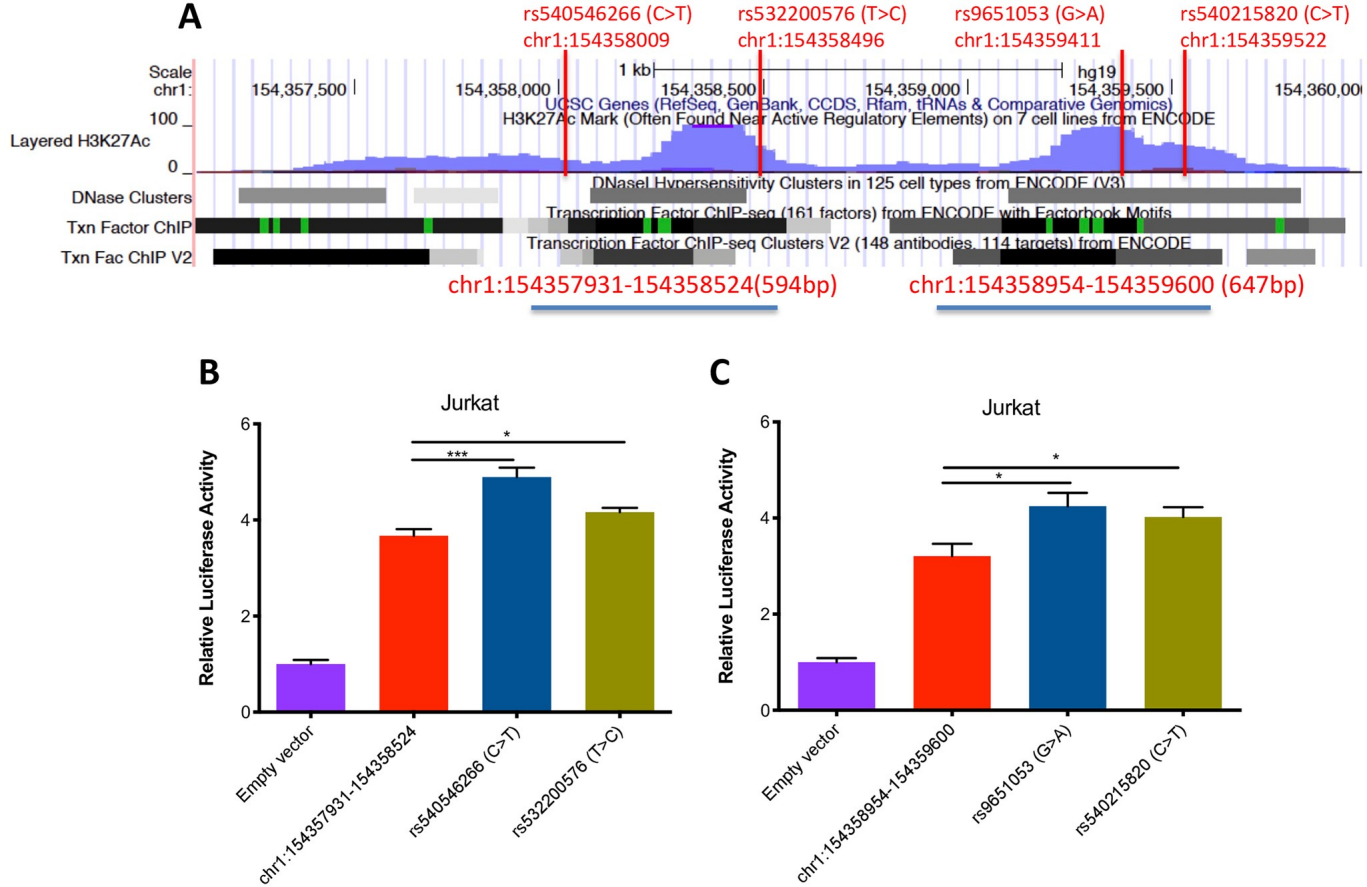
The effects of JIA-associated genetic variants (*IL6R* locus) on enhancer activity in Jurkat T cells. A. Two constructs (chr1:154357931–15435852, 594bp and chr1:154358954–154359600,647bp) within an intergenic region upstream of the *IL6R* gene were used as template to generate 4 enhancer genetic variants. These SNPs were either identified as having high likelihood of having regulatory effects using the SNiPA software program [40] (rs540546266 [C>T], rs532200576 [T>C], rs540215820 [C>T]), or were identified in the Cincinnati Children’s Hospital JIA genotyping cohort (rs9651053, [G>A]). B and C. The results are from 594bp and 647 constructs, respectively. The second bar represents a construct containing the common allele. The third and fourth bars show the effects of JIA-associated genetic variants on enhancer activity in Jurkat T cells. The empty vector (negative control) is the basic pGL4.23 vector (which contains the SV40 promoter but is inefficient at driving luciferase expression). Data are represented as mean ± SEM of 6 biological replicates, and p values are calculated using Student’s t test. *p≤0.05; ***p≤0.001.

We next tested the effects of 4 variants within the *IL6R* intergenic enhancer. These SNPs were either identified as having high likelihood of having regulatory effects using the SNiPA software program [40] (rs540546266 [C>T], rs532200576 [T>C], rs540215820 [C>T]), or were identified in the Cincinnati Children’s Hospital JIA genotyping cohort (rs9651053, [G>A]) as being more common in children with JIA (n = 2,751) than in healthy controls (n = 15,8885). We prepared two constructs spanning the region of interest and tested each construct for luciferase production. Both constructs (chr1:154357931–154358524, 594bp and chr1:154358954–154359600, 647bp, Fig 7A) with brisk enhancer activity that was observable in Jurkat T cells. These results are summarized in Fig 7B and 7C. All 4 of these variants significantly increased enhancer function in reporter assays compared with the common allele (Fig 7B and 7C). Three other variants identified on SNiPA (rs528368588 [A>G], rs538144605 [G>A], and rs141269130 [C>T]) as well as one variant identified in the Cincinnati data (rs6427627 [T>C]) showed no significant effect (p>0.05) compared to the common allele (data not shown).

**Fig 8:**
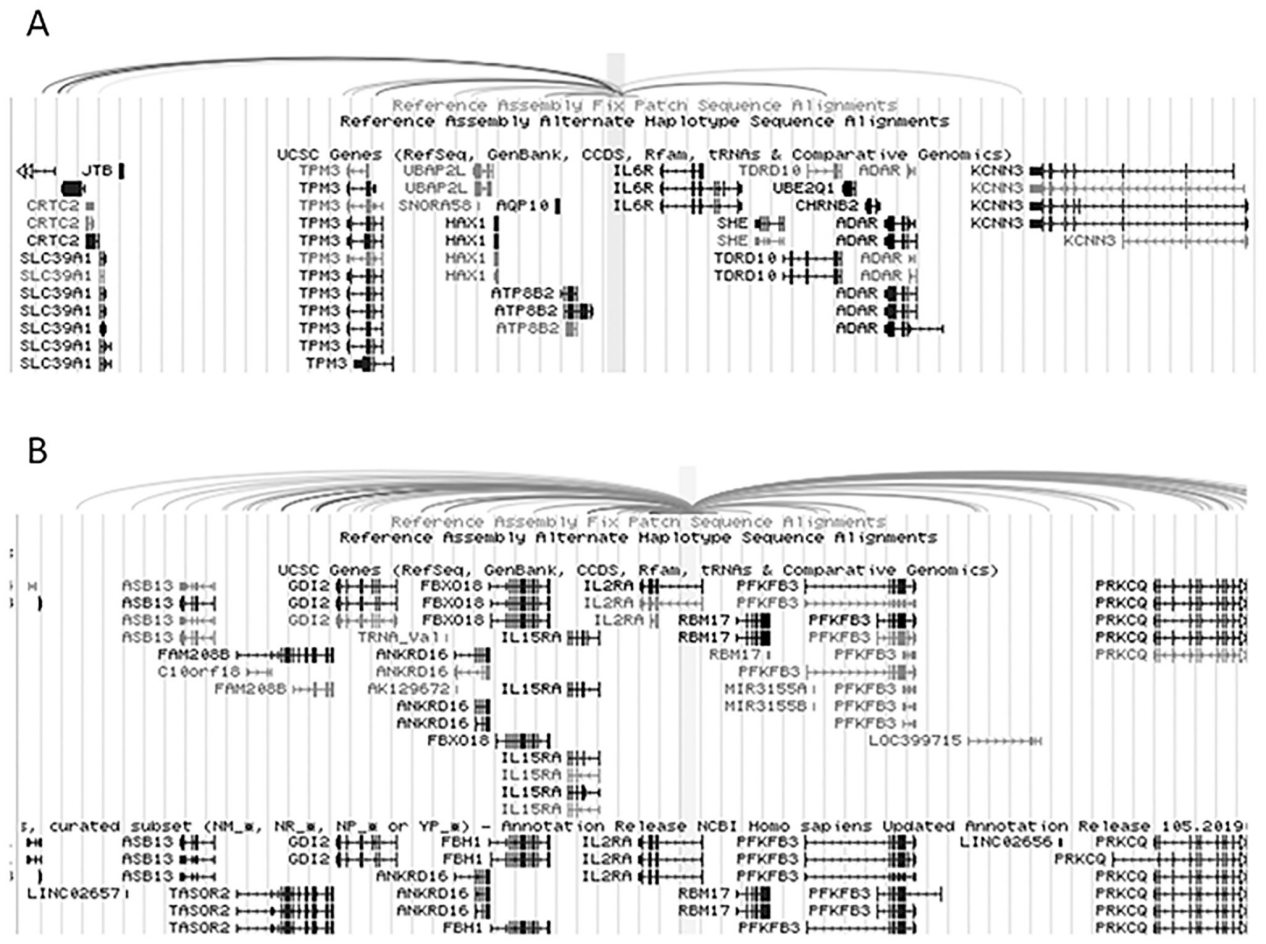
Genome browser screen shot showing results from promoter capture HiC data in unfractionated human peripheral blood CD4+ T cells [25]. H3K27ac-marked regions within the *IL6R* (A) and *IL2RA* (B) risk haplotypes form contact loops with multiple genes, including genes not on the risk haplotypes.

When we queried H3K27ac-based promoter capture HiC data available on the 3D Genome Browser [18, 31], we found multiple genes contacting the identified *IL2RA* and *IL6R* enhancers, as shown in Fig 8. For *IL6R*, promoter capture HiC demonstrated contact points with multiple genes (e.g., *TPN3*, *KCNN3*) not on the JIA haplotype (Fig 8A). This was also true of the *IL2RA* locus (Fig 8B). These finding support the idea that genetic variants that contribute to disease risk, in many instances, exert their effects on genes that are not on the actual risk haplotypes [41].

## Discussion

Genome-wide association studies and genetic fine mapping studies have provided the field of pediatric rheumatology with a treasure trove of information regarding genetic risk for JIA. However, these studies have still not identified the actual causal SNPs, i.e., the genetic variants that exert the biological effects that confer risk, the genomic processes altered by genetic variants, or the target genes whose function/expression is altered by the causal variants. This is because the tag SNPs used to identify risk loci on GWAS are in linkage disequilibrium with dozens, sometimes hundreds or thousands, of other variants, most of which are likely to be neutral with respect to disease risk. The JIA risk haplotypes may extend over regions >100,000 bp and typically contain multiple genes as well as functional regulatory elements. We have recently shown that the JIA risk haplotypes are highly enriched for H3K4me1/H3K27ac marks, epigenetic signatures that are typically associated with enhancer function, and that many of the JIA haplotypes contain multiple H3K4me1/H3K27ac ChIPseq peaks [4, 5]. Since enhancers function by looping transcription factor-rich chromatin segments to promoters, we sought further evidence that genetic risk in JIA impinges on enhancer function by mining publically available HiC data. We specifically looked for evidence of contact between H3K4me1/H3K27ac-marked regions within established JIA haplotypes and the promoters of immunologically relevant genes.

We found strong evidence from HiC data that the H3K4me1/H3K27ac-marked regions within the JIA haplotypes are physically associated with promoters of multiple genes. Furthermore, these interactions could be seen in both lymphoid and myeloid cell lines, a finding consistent with other experimental evidence that suggests complex interactions between innate and adaptive immunity in JIA [42, 43]. HiC findings were supported by H3K27ac-based promoter capture HiC data from CD4+ T cells, where we visualized direct contact between H3K27ac-marked regions on the JIA haplotypes and the promoters of immune-related genes (e.g., Fig 8). GO analysis of the genes in contact within the HiC-defined TADs (Fig 3) revealed cogent patterns of genes associated with lymphocyte activation and proliferation as well as signaling processes (e.g., MAP kinase signaling) important in both innate and adaptive immunity.

It is important to note that enhancer function can’t be determined on the basis of chromatin structure/features alone. Thus, to corroborate our analyses of HiC data, we experimentally tested enhancer function within the JIA-associated *IL2RA* and *IL6R* loci. *IL2RA* encodes for the alpha chain of the IL2 receptor (CD25). *IL2RA* was identified as a JIA risk locus through a candidate gene approach [22] and validated on genetic fine mapping studies [25]. Because of the importance of IL2 in a broad spectrum of adaptive immune functions as well as particular interest in CD25+FoxP3+ regulatory T cells in JIA [44], it has been assumed that variants in this locus must impact *IL2RA* expression and adaptive immune function. *IL6R* encodes the alpha subunit of the IL6 receptor. IL6 is a known mediator of the pathogenic processes associated with a broad range of autoimmune and inflammatory diseases, and IL6R is the target of the therapeutic agent, tocilizumab, which has been shown to be efficacious in treating multiple autoimmune diseases, including JIA [45].

We identified enhancer function within the first intron of *IL2RA*, and, furthermore, demonstrated that genetic variants that we had previously identified on WGS of patients with polyarticular JIA [39] attenuate that function. In addition, we identified multiple JIA-associated genetic variants that altered the efficiency of the intergenic enhancer upstream of the *IL6R* gene. This is the first instance, as far as we are aware, of the identification of specific, non-coding functions that are altered by JIA-associated genetic variants. We note, as well, that although many intronic enhancers regulate the transcriptional efficiency of the genes in which they are located [46], the HiC data from the *IL2RA* locus (see Table 2) is consistent with the hypothesis that there are other genes regulated by this enhancer (immune cell enhancers often regulate more than one gene). Similarly, both HiC (Table 2) and promoter capture HiC data (Fig 8B) support the idea that the *IL6R* intergenic enhancer may regulate multiple genes, including genes not actually on the JIA risk haplotypes. This concept \has recently been demonstrated directly for the risk haplotypes associated with systemic lupus [41].

There are obvious limitations to using data from cell lines to make inferences about the genetics of human diseases. The chromatin architecture of the cell lines is unlikely to replicate that of the relevant primary human cells is every respect. To compensate for these limitations, we used the chromatin architecture from primary human neutrophils and CD4+ T cells to inform the regions we chose to query [4, 5]. Furthermore, we corroborated 3D data from HiC studies in cell lines with promoter capture HiC data from primary human CD4+ T cells [31]. Thus, while these studies carry all the limitations of data generated in cell lines, they are supported by the findings in primary cells.

Unequivocally identifying target genes, and the cells within which genetic effects are exerted more strongly, remains an important task for the field, and advances in genomic technologies are likely to facilitate this effort. For example, Gasperini et al have recently shown the feasibility of using genome-wide screens to identify the target genes of enhancers [47]. The screening process uses CRISPR-based technologies to alter the post-translational modifications of histones, an approach that has been shown to be quite specific and relatively free of off-target effects [48]. The bigger challenge may be to identify the specific immune cells in which genetically-mediated effects on transcription (e.g., by modification of enhancer function) are exerted. It is now becoming clear that genetic effects on gene expression in immune cells are exerted in a highly cell subtype-specific way; this has been shown in both normal cells and those from patients with autoimmune disease [49]. Thus, teasing out the disease-relevant effects of genetically-modified enhancers will also likely require single-cell sequencing approaches on genotyped populations.

Taken together, this study shows the importance of extending our investigations into genetic risk for JIA beyond the singular focus on the “nearest gene” to the tag SNPs [50]. The tag SNPs and the extended haplotypes containing them are themselves components of complex 3-dimensional chromatin structures that show both overlapping and cell-type-specific features in the different immune cell types. These larger chromatin structures encompass multiple genes that are immunologically relevant and potential targets of therapy. We contend that the next important task for the field will be a systematic interrogation of these regions to identify relevant, risk-conferring variants within H3K4me1/H3K27ac-marked regions. It is also essential that we identify the target genes whose expression is altered (augmented or attenuated) by genetically altered enhancers. Having such data will be essential if we are to deliver on the promise of precision medicine to improve the lives of patients.

## Supporting information

S1 Table (PDF)

S2 Table (PDF)

S3 Table (PDF)
