# Supplementary material for: Broadening our understanding of the genetics of Juvenile Idiopathic Arthritis (JIA): Interrogation of three dimensional chromatin structures and genetic regulatory elements within JIA-associated risk loci
Source: PLoS One. 2020 Jul 30;15(7):e0235857. doi: 10.1371/journal.pone.0235857 (PMC7392255; doi:10.1371/journal.pone.0235857)
Supplement: S1 Table — (PDF) [file pone.0235857.s001.pdf]

Table S1

Enhancer	Location of LD block	GM12878 Genes	K562 Genes	T-Cell Genes	THP-1 Macrophage Genes
SH2B3	chr12: 111884608 – 111932800	BRAP<br>ATXN2-AS1<br>ATXN2<br>SH2B3<br>PHETA1<br>CUX2	BRAP<br>ATXN2-AS<br>ATXN2<br>SH2B3<br>PHETA1<br>CUX2	PTPN11<br>RPL6<br>HECTD4<br>MIR6861<br>TRAFD1<br>NAA25<br>MIR3657<br>ERP29<br>TMEM116<br>ADAM1A<br>MAPKAPK5<br>NR_152605<br>NR_015404<br>ALDH2<br>MIR6761<br>ACAD10<br>BRAP<br>ATXN2<br>ATXN2-AS<br>SH2B3<br>PHETA1<br>CUX2	ATXN2<br>SH2B3<br>PHETA1<br>CUX2<br>ATXN2-AS<br>BRAP<br>ACAD10
NAA25	chr12: 112486818 – 112906415	RPL6<br>HECTD4<br>MIR6861<br>TRAFD1<br>NAA25<br>MIR3657<br>ERP29<br>TMEM116	NAA25<br>HECTD4<br>MIR6861<br>TRAFD1<br>MIR3657<br>ERP29<br>TMEM116<br>ADAM1A<br>MAPKAPK5<br>NR_152605<br>NR_015404	PTPN11<br>RPL6<br>HECTD4<br>MIR6861<br>TRAFD1<br>NAA25<br>MIR3657<br>ERP29<br>TMEM116<br>ADAM1A<br>MAPKAPK5<br>NR_152605<br>NR_015404<br>ALDH2<br>MIR6761<br>ACAD10<br>BRAP<br>ATXN2<br>ATXN2-AS<br>SH2B3<br>PHETA1<br>CUX2	NR_015404<br>NR_152605<br>MAPKAPK5<br>ADAM1A<br>TMEM116<br>ERP29<br>NAA25<br>MIR3657<br>TRAFD1<br>HECTD4<br>MIR6861<br>RPL6<br>ALDH2<br>MIR6761<br>ACAD10<br>BRAP<br>ATXN2-AS<br>ATXN2
PTPN2	chr18: 12821903 – 12880206	PSMG2<br>LINC01882<br>PTPN2<br>SEH1L<br>CEP192<br>CEP76<br>SPIRE1<br>NR_136514<br>PRELID3A	PTPN2<br>SEH1L<br>CEP192<br>LINC01882<br>PSMG2<br>CEP76<br>SPIRE1<br>NR_136514<br>PRELID3A	LINC01882<br>PTPN2<br>PSMG2<br>CEP76<br>SEH1L<br>CEP192<br>LDLRAD4<br>C18orf15<br>LDLRAD4-AS1<br>MIR5190<br>MIR4526<br>FAM210A<br>RNMT<br>SPIRE1<br>NR_136514<br>PRELID3A	SEH1L<br>PTPN2<br>LINC01882<br>PSMG2<br>CEP76<br>CEP192
STAT4	chr2: 191900449 – 191935804	STAT4<br>MYO1B	MYO1B<br>STAT4	MYO1B<br>STAT4	MYO1B<br>STAT4

		NR_136318<br>STAT1<br>GLS			NR_136318<br>STAT1<br>GLS
MIF	chr22: 24234493 –<br>24237862	SLC2A11<br>MIF<br>MIF-AS1<br>GSTT2B<br>DDTL<br>DDT<br>GSTT4<br>LOC391322<br>GSTT1-AS1<br>GSTT1<br>GSTTP2<br>CABIN1<br>SUSD2<br>GGT5<br>POM121L9P<br>SPECC1L<br>DERL3<br>SMARCB1<br>MMP11<br>CHCHD10<br>VPREB3<br>ZNF70<br>GUSBP11<br>RGL4<br>DRICH1<br>IGLL1	SPECC1L<br>POM121L9P<br>GGT5<br>SUSD2<br>CABIN1<br>GSTTP2<br>GSTT1<br>GSTT1-AS1<br>LOC391322<br>GSTT4<br>GSTT2B<br>DDT<br>DDTL<br>MIF<br>MIF-AS1<br>SLC2A11<br>DERL3<br>SMARCB1<br>MMP11<br>CHCHD10<br>ZNF70<br>GUSBP11<br>RGL4<br>DRICH1<br>IGLL1	GSTT2B<br>MIF<br>MIF-AS1<br>SLC2A11<br>DERL3<br>SMARCB1<br>MMP11<br>CHCHD10<br>VPREB3<br>ZNF70<br>GUSBP11<br>RGL4<br>DRICH1<br>IGLL1	SPECC1L<br>POM121L9P<br>GGT5<br>SUSD2<br>CABIN1<br>GSTTP2<br>GSTT1<br>GSTT1-AS1<br>LOC391322<br>GSTT4<br>GSTT2B<br>DDT<br>DDTL<br>MIF<br>MIF-AS1<br>SLC2A11<br>DERL3<br>SMARCB1<br>MMP11<br>CHCHD10<br>VPREB3<br>ZNF70<br>GUSBP11<br>RGL4<br>DRICH1<br>IGLL1
TIMMDC1	chr3: 119125202 –<br>119243934	COX17<br>POPDC2<br>PLA1A<br>ADPRH<br>CD80<br>TIMMDC1<br>POGLUT1<br>TMEM39A<br>ARHGAP31<br>NR_046748<br>B4GALT4-AS1<br>B4GALT4	COX17<br>POPDC2<br>PLA1A<br>ADPRH<br>CD80<br>TIMMDC1<br>POGLUT1<br>TMEM39A<br>ARHGAP31<br>NR_046748<br>B4GALT4-AS1<br>B4GALT4<br>UPK1B<br>C3orf30<br>IGSF11<br>IGSF11-AS1	COX17<br>POPDC2<br>PLA1A<br>ADPRH<br>CD80<br>TIMMDC1<br>POGLUT1<br>TMEM39A<br>ARHGAP31<br>NR_046748<br>B4GALT4-AS1<br>B4GALT4<br>UPK1B<br>C3orf30<br>IGSF11	COX17<br>POPDC2<br>PLA1A<br>CD80<br>TIMMDC1<br>POGLUT1<br>TMEM39A<br>ARHGAP31<br>NR_046748<br>B4GALT4-AS1<br>B4GALT4<br>UPK1B<br>C3orf30<br>IGSF11<br>IGSF11-AS1
CCR1	chr3: 46253650 –<br>46350716	NR_125406<br>CCR5<br>CCR2<br>CCR3<br>CCR1<br>XCR1<br>FYCO1<br>CXCR6<br>CCR9<br>LZTFL1<br>SLC6A20	CCR5<br>CCR2<br>NR_125406<br>CCR3<br>CCR1	LTF<br>LINC02009<br>NR_125406<br>CCRL2<br>CCR5<br>CCR2<br>CCR3<br>CCR1<br>RTP3<br>LRRC2<br>TDGF1<br>LRRC2-AS1<br>FAM240A<br>ALS2CL<br>TMIE<br>PRSS50<br>PRSS46P<br>FYCO1<br>XCR1<br>CXCR6	CCR5<br>NR_125406<br>CCR2<br>CCR3<br>CCR1<br>CCRL2<br>LINC02009<br>LTF<br>XCR1<br>FYCO1<br>CXCR6

				LZTFL1<br>CCR9<br>SLC6A20<br>SACM1L<br>PRSS45	
ADAD1	chr4: 123141054 – 123548068	IL2<br>ADAD1<br>KIAA1109<br>TRPC3	IL2<br>ADAD1<br>KIAA1109<br>TRPC3	IL21-AS1<br>IL21<br>IL2<br>ADAD1<br>KIAA1109<br>TRPC3<br>BBS7<br>CCNA2<br>EXOSC9<br>PP12613<br>TMEM155	IL2<br>ADAD1<br>KIAA1109<br>IL21<br>IL21-AS1<br>CETN4P<br>BBS12<br>FGF2<br>NUDT6<br>SPATA5<br>TRPC3
WISP3	chr6: 112359543 – 112448654	WISP3<br>LINC02527<br>FYN<br>TUBE1<br>FAM229B<br>LAMA4<br>TRAF3IP2<br>NR_034110<br>NR_034111<br>REV3L<br>MFSD4B	WISP3<br>FYN<br>LINC02527<br>TUBE1<br>FAM229B<br>LAMA4	WISP3<br>FYN<br>LINC02527	LAMA4<br>FAM229B<br>TUBE1<br>WISP3<br>LINC02527<br>FYN
JAZF1	chr7: 28152193 – 28243473	JAZF1<br>JAZF1-AS1<br>CREB5	CREB5<br>JAZF1-AS1<br>JAZF1<br>TAX1BP1<br>HIBADH<br>TSL<br>EVX1<br>HOTTIP<br>HOXA13<br>HOXA11-AS<br>HOXA11	TAX1BP1<br>JAZF1<br>HIBADH<br>TSL<br>EVX1<br>HOXA13<br>HOTTIP<br>JAZF1-AS1<br>CREB5	JAZF1-AS1<br>JAZF1
TRAF1-C5	chr9: 123636121 – 123723351	C5<br>C5-OT1<br>TRAF1<br>PHF19<br>CUTALP<br>PSMD5<br>B3GNT10<br>FBXW2<br>RAB14<br>CNTRL<br>GSN<br>GSN-AS1<br>STOM	FBXW2<br>B3GNT10<br>PSMD5<br>CUTALP<br>PHF19<br>TRAF1<br>C5-OT1<br>C5	FBXW2<br>B3GNT10<br>PSMD5<br>CUTALP<br>PHF19<br>TRAF1<br>C5-OT1<br>C5<br>CNTRL<br>RAB14<br>GSN	C5-OT1<br>TRAF1<br>PHF19<br>CUTALP<br>C5<br>PSMD5<br>B3GNT10<br>FBXW2<br>MEGF9
PHTF1-RSBN	chr1: 114303808–114377568	OLFML3<br>HIPK1<br>HIPK1-AS1<br>DCLRE1B<br>AP4B1<br>BCL2L15<br>AP4B1-AS1<br>PTPN22<br>RSBN1<br>PHTF1	MAGI3<br>RSBN1<br>PHTF1<br>PTPN22<br>AP4B1-AS1<br>BCL2L15<br>AP4B1<br>DCLRE1B<br>HIPK1-AS1<br>HIPK1	OLFML3<br>HIPK1<br>HIPK1-AS1<br>DCLRE1B<br>AP4B1<br>BCL2L15<br>AP4B1-AS1<br>PTPN22<br>RSBN1<br>PHTF1<br>MAGI3<br>LRIG2<br>LOC643441	OLFML3<br>HIPK1<br>HIPK1-AS1<br>DCLRE1B<br>AP4B1<br>BCL2L15<br>AP4B1-AS1<br>PTPN22<br>RSBN1<br>PHTF1<br>MAGI3<br>LOC643441
IL6R	chr1: 154291718–	SHE	IL6R	None	IL6R

	154379369	IL6R<br>IL6R-AS1<br>ATP8B2<br>UBE2Q1<br>UBE2Q1-AS1<br>TDRD10<br>AQP10<br>HAX1<br>UBAP2L<br>SNORA58B<br>C1orf43<br>C1orf189<br>MIR190B<br>TPM3<br>NUP210L<br>MIR5698<br>RPS27	IL6R-AS1<br>ATP8B2<br>SHE<br>TDRD10<br>UBE2Q1<br>UBE2Q1-AS1<br>AQP10<br>HAX1<br>UBAP2L<br>SNORA58B<br>C1orf43<br>C1orf189<br>MIR190B<br>TPM3		IL6R-AS1<br>ATP8B2<br>AQP10<br>UBE2Q1<br>UBE2Q1-AS1<br>TDRD10<br>SHE<br>HAX1<br>UBAP2L<br>SNORA58B<br>TPM3<br>C1orf43<br>C1orf189<br>MIR190B
IL2RA	chr10: 6078553–6097283	ASB13<br>FAM208B<br>GDI2<br>ANKRD16<br>FBH1<br>IL15RA<br>IL2RA<br>RMB17<br>NR_134491<br>CALML3<br>CALML3-AS1<br>CALML5<br>NET1<br>PFKFB3<br>MIR3155B<br>LOC399715<br>LOC399716<br>PRKCQ	IL2RA<br>IL15RA<br>RBM17<br>PFKFB3<br>MIR3155B<br>LOC399715<br>LOC399716<br>PRKCQ<br>ANKRD16<br>FAM208B<br>ASB13<br>GDI2<br>FBH1	IL2RA<br>RBM17<br>PFKFB3<br>LOC399715<br>MIR3155B<br>LOC399716<br>PRKCQ	IL15RA<br>IL2RA<br>RBM17<br>PFKFB3<br>MIR3155B<br>LOC399715<br>LOC399716<br>PRKCQ
FAS	chr10: 90759613–90764891	CH25H<br>LIPA<br>MIR4679-1<br>FAS<br>FAS-AS1<br>ACTA2<br>ACTA2-AS1<br>STAMBPL1<br>ANKRD22<br>LIPM<br>LIPN<br>LIPK<br>LIPF<br>LIPJ<br>RNLS	LIPM<br>ANKRD22<br>STAMBPL1<br>LIPA<br>CH25H<br>MIR4679-1<br>FAS-AS1<br>ACTA2<br>ACTA2-AS1<br>FAS	LIPA<br>CH25H<br>MIR4679-1<br>FAS<br>FAS-AS1<br>ACTA2<br>ACTA2-AS1<br>STAMBPL-1<br>ANKRD22<br>LIPM	FAS<br>FAS-AS1<br>ACTA2<br>ACTA2-AS1<br>MIR4679-1<br>CH25H<br>LIPA<br>STAMBPL1<br>ANKRD22<br>LIPM
ATXN2	chr12: 111884608–111932800	ATXN2<br>SH2B3<br>ATXN2 – AS<br>BRAP	BRAP<br>ATXN2<br>ATXN2-AS<br>SH2B3<br>PHETA1<br>CUX2	PTPN11<br>RPL6<br>HECTD4<br>MIR6861<br>TRAFD1<br>NAA25<br>MIR3657<br>ERP29<br>TMEM116<br>ADAM1A<br>MAPKAPK5<br>NR_015404<br>NR_152605<br>MIR6761<br>ALDH2<br>ACAD10	ATXN2<br>SH2B3<br>PHETA1<br>CUX2<br>ATXN2-AS<br>BRAP<br>ACAD10

				BRAP<br>ATXN2<br>ATXN-AS<br>SH2B3<br>PHETA1<br>CUX2<br>MIR6760	
ZFP36L-RAD51B	chr14: 69250891–69260588	ZFP36L1<br>RAD51B<br>NR_135816	ZFP36L1<br>RAD51B<br>NR_135816	ZFP36L1<br>RAD51B<br>NR_135816	ZFP36L1<br>RAD51B<br>NR_135816
LNPEP	chr5: 96220087–96373750	RIOK2<br>LIX1<br>LNPEP<br>ERAP2<br>ERAP1<br>CAST<br>NR_130776	ERAP2<br>LNPEP<br>LIX1<br>RIOK2<br>ERAP1<br>CAST<br>NR_130776	RIOK2<br>LIX1<br>LNPEP<br>ERAP2<br>ERAP1<br>CAST	RIOK2<br>LIX1<br>LNPEP<br>ERAP2<br>ERAP1<br>CAST<br>NR_130776<br>PCSK1
C5orf56-IRF1	chr5: 131813219–131832514	IRF1<br>C5orf56<br>SLC22A5<br>MIR3936<br>MIR3936HG<br>SLC22A4<br>PDLIM4<br>P4HA2<br>MIR6830<br>P4HA2-AS1<br>CSF2<br>IL3<br>IL5<br>RAD50<br>TH2LCRR<br>IL13<br>IL4<br>NR_134248<br>KIF3A<br>CCNI2<br>SEPT8<br>SOWAHA<br>SHROOM1<br>GDF9<br>UQCRQ<br>LEAP2<br>AFF4	IRF1<br>IL5<br>RAD50<br>TH2LCRR<br>IL13<br>IL4<br>NR_134248<br>KIF3A<br>CCNI2<br>SEPT8<br>SOWAHA<br>SHROOM1<br>UQCRQ<br>GDF9<br>LEAP2<br>AFF4<br>C5orf56<br>IRF1<br>SLC22A5<br>MIR3936<br>MIR3936HG<br>SLC22A4<br>PDLIM4<br>P4HA2<br>MIR6830<br>P4HA2-AS1<br>CSF2	SHROOM1<br>SOWAHA<br>CCNI2<br>SEPT8<br>KIF3A<br>NR_134248<br>IL4<br>IL13<br>TH2LCRR<br>RAD50<br>IL5<br>IRF1<br>C5orf56<br>SLC22A5	SLC22A5<br>C5orf56<br>IRF1<br>IL5<br>RAD50<br>TH2LCRR<br>IL13<br>IL4<br>NR_134248<br>KIF3A<br>CCNI2<br>SEPT8<br>SOWAHA<br>SHROOM1<br>GDF9<br>UQCRQ<br>LEAP2<br>MIR3936<br>MIR3936HG<br>SLC22A4<br>PDLIM4<br>P4HA2<br>MIR6830<br>P4HA2-AS1<br>CSF2<br>IL3
