# Supplementary material for: Broadening our understanding of the genetics of Juvenile Idiopathic Arthritis (JIA): Interrogation of three dimensional chromatin structures and genetic regulatory elements within JIA-associated risk loci
Source: PLoS One. 2020 Jul 30;15(7):e0235857. doi: 10.1371/journal.pone.0235857 (PMC7392255; doi:10.1371/journal.pone.0235857)
Supplement: S2 Table — (PDF) [file pone.0235857.s002.pdf]

Table S2 – THP1 cells

GO Term	Description	P-value	Number of genes
GO:0009967	positive regulation of signal transduction	3.02E-07	34
GO:0019221	cytokine-mediated signaling pathway	6.20E-07	28
GO:0010647	positive regulation of cell communication	1.60E-06	34
GO:0023056	positive regulation of signaling	2.97E-06	34
GO:1902533	positive regulation of intracellular signal transduction	3.25E-06	24
GO:0042509	regulation of tyrosine phosphorylation of STAT protein	5.29E-06	10
GO:0050731	positive regulation of peptidyl-tyrosine phosphorylation	6.02E-06	12
GO:0035556	intracellular signal transduction	8.56E-06	29
GO:0050730	regulation of peptidyl-tyrosine phosphorylation	9.19E-06	14
GO:1903706	regulation of hemopoiesis	9.67E-06	16
GO:1904892	regulation of STAT cascade	2.36E-05	11
GO:0032946	positive regulation of mononuclear cell proliferation	2.36E-05	11
GO:0070665	positive regulation of leukocyte proliferation	2.36E-05	11
GO:0050671	positive regulation of lymphocyte proliferation	2.36E-05	11
GO:0046425	regulation of JAK-STAT cascade	2.36E-05	11
GO:0070098	chemokine-mediated signaling pathway	2.36E-05	9
GO:0007204	positive regulation of cytosolic calcium ion concentration	2.91E-05	12
GO:0001934	positive regulation of protein phosphorylation	3.31E-05	25
GO:0031401	positive regulation of protein modification process	3.67E-05	27
GO:1902531	regulation of intracellular signal transduction	3.79E-05	31
GO:0042327	positive regulation of phosphorylation	4.77E-05	26
GO:0051480	regulation of cytosolic calcium ion concentration	5.72E-05	12
GO:0000165	MAPK cascade	5.72E-05	12
GO:0010562	positive regulation of phosphorus metabolic process	6.41E-05	26
GO:0045937	positive regulation of phosphate metabolic process	6.41E-05	26

GO:0001932	regulation of protein phosphorylation	8.57E-05	29
GO:0009966	regulation of signal transduction	9.14E-05	42
GO:0042531	positive regulation of tyrosine phosphorylation of STAT protein	1.02E-04	8
GO:0023014	signal transduction by protein phosphorylation	1.06E-04	12
GO:1904894	positive regulation of STAT cascade	1.54E-04	9
GO:0046427	positive regulation of JAK-STAT cascade	1.54E-04	9
GO:1902105	regulation of leukocyte differentiation	1.87E-04	12
GO:0019220	regulation of phosphate metabolic process	2.33E-04	33
GO:0051174	regulation of phosphorus metabolic process	2.33E-04	33
GO:0006954	inflammatory response	2.45E-04	18
GO:0051247	positive regulation of protein metabolic process	2.77E-04	30
GO:0006875	cellular metal ion homeostasis	2.82E-04	13
GO:0055065	metal ion homeostasis	2.82E-04	13
GO:0006874	cellular calcium ion homeostasis	3.16E-04	12
GO:0055074	calcium ion homeostasis	3.16E-04	12
GO:0032944	regulation of mononuclear cell proliferation	3.16E-04	12
GO:0070663	regulation of leukocyte proliferation	3.16E-04	12
GO:0072507	divalent inorganic cation homeostasis	3.16E-04	12
GO:0072503	cellular divalent inorganic cation homeostasis	3.16E-04	12
GO:0050670	regulation of lymphocyte proliferation	3.16E-04	12
GO:0051249	regulation of lymphocyte activation	3.79E-04	16
GO:0042325	regulation of phosphorylation	4.04E-04	31
GO:0051251	positive regulation of lymphocyte activation	4.45E-04	13
GO:0060326	cell chemotaxis	5.13E-04	12
GO:0008284	positive regulation of cell proliferation	5.49E-04	21
GO:0045619	regulation of lymphocyte differentiation	5.72E-04	8
GO:0042102	positive regulation of T cell proliferation	5.72E-04	8
GO:0019722	calcium-mediated signaling	5.72E-04	8

GO:0007166	cell surface receptor signaling pathway	6.52E-04	37
GO:0048584	positive regulation of response to stimulus	6.52E-04	37
GO:0006468	protein phosphorylation	6.52E-04	18
GO:0030890	positive regulation of B cell proliferation	6.75E-04	6
GO:1903707	negative regulation of hemopoiesis	6.75E-04	6
GO:0031399	regulation of protein modification process	6.90E-04	32
GO:0032270	positive regulation of cellular protein metabolic process	7.78E-04	27
GO:0002683	negative regulation of immune system process	8.29E-04	14
GO:0065008	regulation of biological quality	8.81E-04	50
GO:0045580	regulation of T cell differentiation	9.99E-04	7
GO:0050871	positive regulation of B cell activation	9.99E-04	7
