# Supplementary material for: Broadening our understanding of the genetics of Juvenile Idiopathic Arthritis (JIA): Interrogation of three dimensional chromatin structures and genetic regulatory elements within JIA-associated risk loci
Source: PLoS One. 2020 Jul 30;15(7):e0235857. doi: 10.1371/journal.pone.0235857 (PMC7392255; doi:10.1371/journal.pone.0235857)
Supplement: S3 Table — (PDF) [file pone.0235857.s003.pdf]

Table S3 – K562 cells

GO Term	Description	P-value	Number of genes
GO:0019221	cytokine-mediated signaling pathway	2.12E-06	21
GO:0009967	positive regulation of signal transduction	5.49E-06	31
GO:0032944	regulation of mononuclear cell proliferation	1.38E-05	11
GO:0050670	regulation of lymphocyte proliferation	1.38E-05	11
GO:0032946	positive regulation of mononuclear cell proliferation	1.58E-05	10
GO:0070665	positive regulation of leukocyte proliferation	1.58E-05	10
GO:0050671	positive regulation of lymphocyte proliferation	1.58E-05	10
GO:0006954	inflammatory response	1.78E-05	15
GO:0010647	positive regulation of cell communication	2.04E-05	31
GO:0023056	positive regulation of signaling	2.04E-05	31
GO:0060326	cell chemotaxis	3.55E-05	9
GO:0070663	regulation of leukocyte proliferation	4.51E-05	11
GO:0030890	positive regulation of B cell proliferation	4.80E-05	5
GO:0048584	positive regulation of response to stimulus	4.81E-05	35
GO:0051249	regulation of lymphocyte activation	5.82E-05	15
GO:0006955	immune response	6.27E-05	21
GO:1904892	regulation of STAT cascade	7.15E-05	9
GO:0046425	regulation of JAK-STAT cascade	7.15E-05	9
GO:0042509	regulation of tyrosine phosphorylation of STAT protein	7.88E-05	8
GO:0050920	regulation of chemotaxis	7.88E-05	8
GO:0006935	chemotaxis	1.03E-04	10
GO:0042330	taxis	1.03E-04	10
GO:0050865	regulation of cell activation	1.06E-04	16
GO:0080134	regulation of response to stress	1.23E-04	27
GO:1902533	positive regulation of intracellular signal transduction	1.33E-04	21

GO:0050730	regulation of peptidyl-tyrosine phosphorylation	1.33E-04	12
GO:0009966	regulation of signal transduction	1.36E-04	40
GO:0002688	regulation of leukocyte chemotaxis	1.42E-04	6
GO:0001959	regulation of cytokine-mediated signaling pathway	1.42E-04	6
GO:0050871	positive regulation of B cell activation	1.42E-04	6
GO:0060759	regulation of response to cytokine stimulus	1.42E-04	6
GO:0002694	regulation of leukocyte activation	1.64E-04	15
GO:0001934	positive regulation of protein phosphorylation	1.71E-04	22
GO:0032103	positive regulation of response to external stimulus	1.73E-04	7
GO:0050864	regulation of B cell activation	1.73E-04	7
GO:0002683	negative regulation of immune system process	1.94E-04	13
GO:0001819	positive regulation of cytokine production	1.94E-04	13
GO:0002376	immune system process	2.07E-04	32
GO:0042327	positive regulation of phosphorylation	2.11E-04	23
GO:0030888	regulation of B cell proliferation	2.56E-04	5
GO:0050731	positive regulation of peptidyl-tyrosine phosphorylation	2.82E-04	10
GO:0051240	positive regulation of multicellular organismal process	2.91E-04	28
GO:0051251	positive regulation of lymphocyte activation	2.95E-04	12
GO:1902105	regulation of leukocyte differentiation	2.99E-04	11
GO:1902531	regulation of intracellular signal transduction	3.46E-04	28
GO:0002407	dendritic cell chemotaxis	3.55E-04	4
GO:0036336	dendritic cell migration	3.55E-04	4
GO:0050922	negative regulation of chemotaxis	3.55E-04	4
GO:1904894	positive regulation of STAT cascade	3.68E-04	7
GO:0046427	positive regulation of JAK-STAT cascade	3.68E-04	7
GO:0030595	leukocyte chemotaxis	3.76E-04	6
GO:0002696	positive regulation of leukocyte activation	4.24E-04	12
GO:0050867	positive regulation of cell activation	4.24E-04	12

GO:0010562	positive regulation of phosphorus metabolic process	4.72E-04	23
GO:0045937	positive regulation of phosphate metabolic process	4.72E-04	23
GO:0032101	regulation of response to external stimulus	5.37E-04	15
GO:0031347	regulation of defense response	6.31E-04	14
GO:0006952	defense response	6.33E-04	20
GO:0031401	positive regulation of protein modification process	6.49E-04	24
GO:0000165	MAPK cascade	6.65E-04	10
GO:0002700	regulation of production of molecular mediator of immune response	7.06E-04	7
GO:0050868	negative regulation of T cell activation	8.34E-04	6
GO:0070098	chemokine-mediated signaling pathway	8.34E-04	6
GO:1902107	positive regulation of leukocyte differentiation	9.08E-04	8
GO:0045619	regulation of lymphocyte differentiation	9.08E-04	8
GO:0050866	negative regulation of cell activation	9.08E-04	8
GO:0042129	regulation of T cell proliferation	9.08E-04	8
GO:0002684	positive regulation of immune system process	9.85E-04	18
GO:0050900	leukocyte migration	9.86E-04	9
